# Depilatory Wax Burns: Experience and Investigation

**Published:** 2011-05-13

**Authors:** Angela C Chang, Katherine M Watson, Tara L Aston, Marcus JD Wagstaff, John E Greenwood

**Affiliations:** ^a^University of Adelaide Medical School, Adelaide, South Australia; ^b^Adult Burn Service, Royal Adelaide Hospital, Adelaide, South Australia

## Abstract

**Objectives:** To retrospectively collect data on patients with burn injury due to hot depilatory wax. To investigate the effect of varying microwave output power on wax temperature. To determine whether instructions provided by manufacturers allow safe domestic use. **Methods:** Data from the RAH burns database was collected for patients with wax-induced burns between January 1991 and January 2010. Wax temperatures were tested in a pilot study (4 wax products heated in microwave with power outputs of 800 W, 900 W, and 1100 (W) and a definitive study (5 wax products, 3 of each, heated in microwave with power outputs of 800 W, 1000 W, and 1200 (W). A number of different heating regimens were employed and temperatures were recorded using an infrared thermometer. **Results:** Twenty-one patients were studied. Mean age was 26.5 years. The majority of burns were superficial (33.3%) or partial thickness (25.8%). The right hand was most commonly affected (38.1%), the mean total body surface area was 1%. The pilot study revealed an increase in wax temperature with the number of times the wax was heated. During definitive wax temperature testing, the maximum wax temperature recorded was 108.5°C. Seventeen of 60 wax surface temperatures recorded exceeded 90°C, 9 exceeded 100°C. Ninety-three percent of the stirred wax temperatures showed an increase in wax temperature with an increase in microwave power output. **Conclusion:** Microwave-heated hair-removal wax has the potential to reach unsafe temperatures and cause burn injury, even when manufacture's heating instructions are followed. Safe use in domestic setting requires improvements in instructions provided by the manufacturer.

Microwave-heated wax is commonly used for hair removal but has resulted in burn injury. Commercially available waxing products carry instructions for heating but many do not specify the appropriate microwave power and few warn of the potential for burn injury. Modern domestic microwave power outputs range from 800 W to 1200 W. High power settings on different machines can result in significantly different final wax temperatures for the same duration of heating.

A search of the PubMed database returned only 2 articles directly related to burn injury caused by hair removal wax. Zoumaras et al^1^ reviewed 20 patients from 3 hospitals in New South Wales who presented with hair removal wax related burn injury. These burns constituted 1.4% of all burns in New South Wales over the study period (January to November 2006). All burns were superficial to partial thickness, resulting from contact with reheated wax. Of those patients admitted to the Royal North Shore Hospital, 20% required surgical debridement and skin grafting.

Turel-Ermertcan et al^2^ described facial thermal injuries caused by hot hair removal wax in a single patient undergoing isotretinoin treatment for cystic acne. Similar relationships between wax depilation and skin sensitizing acne treatments had previously been described.^3^^-^6 The authors recommend that to prevent future burn injury, beauty centers should be subject to the approval and instruction of dermatologists.

Many beauty centers offer waxing as a method of depilation. Regulatory bodies, including the Government of South Australia, have published guidelines regulating the commercial practice of waxing for hair removal. The South Australian Health Commission publication, *Safe and hygienic practice of skin penetration*,^7^ discusses infection control when working with hair removal wax, but does not provide heating or temperature testing guidelines, warn of the risk of burns, or provide first aid advice for those sustaining burn injury. The *Health Guidelines for Personal Care and Body Art Injuries*, published by the Victorian Government Department of Human Services, includes basic first aid advice for burns.^8^ However, while recommending that wax be heated to 125°C for the purposes of infection control, the risk of burn injury associated with such high temperatures for beautician and client is not discussed.

Over the past 20 years, the Adult Burn Centre at the Royal Adelaide Hospital (RAH) has treated 21 patients with burn injury resulting from microwave-heated hair removal wax products in a domestic setting, with the majority in the last 5 years. This prompted an etiological study focussing on how burns of this nature occur and postulating prevention strategies, because published data is scarce despite the increasing frequency of burn injury.

The aim of this study was to collect data on patients treated at the RAH with burn injury due to hot hair-removal wax; to elucidate incidence, burn site, severity, and mechanism of injury. Then to investigate the effect of varying microwave output power on depilatory wax temperature, to determine whether microwave-heated hair removal wax product instructions are adequate for safe domestic use.

## METHODS

### Cohort analysis

The RAH burns database was reviewed for the period between January 1991 and January 2010, and data collected for all patients treated for burn injury resulting from hot hair removal wax. Twenty-one patients were identified. Data collected included patient gender, age, burn site, burn depth, total body surface area (TBSA) burned, mechanism of injury, length of admission, and surgical intervention.

### Wax temperature testing

Prior to commencing the wax-heating component of the study, a pilot study was conducted. In the pilot study, 4 depilatory wax products were tested, heated according to manufacturer's instructions in microwave with power outputs of 800 W, 900 W, and 1100 W. Measurements from the same 5 regions were taken. Reheating was commenced once the wax was deemed too viscous for use. One bottle of each wax was tested, reused for each microwave, but allowed to cool for more than 16 hours between each test. In the definitive study, 5 commercially available hair removal wax products were selected for testing. They were heated according to the manufacturer's instruction in 3 microwaves of the same brand with power outputs of 800 W, 1000 W, and 1200 W. Three containers of each wax product was purchased so that for each microwave tested a new product was in use. When the manufacturer's instructions did not specify the appropriate microwave power setting, the default (full) power setting was used. Wax temperatures were measured using a precalibrated Testo 830-T1 infrared thermometer (Testo AG, Lenzkirch, Germany) with a measurement range of -30°C to +400°C. Following the initial heating, the wax was allowed to cool while being continuously stirred and the temperature monitored. Reheating was commenced once the wax temperature cooled to 35°C. Wax temperatures were measured before heating, immediately after heating and immediately after reheating from 5 different regions:

1. External side surface of the container
2. External base surface of the container
3. Wax top surface at the perimeter within 1 cm of the edge (before stirring)
4. Wax top surface at the center (before stirring)
5. Wax top surface at the center (after stirring)

### Statistical analysis

The data were compiled and analyzed by using Microsoft Excel 2007 (Microsoft, Redmond, California).

## RESULTS

### Cohort analysis

Between January 1991 and January 2011, 21 patients were treated at the RAH for burn injury resulting from hot microwave-heated hair removal wax (Table 1). Twenty were admitted as inpatients and 1 was treated as an outpatient. All were female. The mean age was 26.5 years with a range of 15 to 54 years. The majority of burns were superficial (33.3%) or partial thickness (25.8%). The hands were involved in 16 of the 21 cases (76%). The right hand was most commonly affected (38.1%) followed by the left hand (14.3%), both hands (9.5%), and left thigh (9.5%). The mean TBSA was 1%. Ten of the 21 patients required debridement under anaesthesia including 2 requiring skin grafts. All cases with a recorded mechanism of injury were due to wax spillage immediately following heating or reheating, most while removing wax from the microwave.

### Wax temperature testing

#### Pilot

The results of the pilot study did not show a strong tendency for the wax temperature to increase with increasing microwave output power, rather, an increase in wax temperature with the number of times the wax was heated. The 800-W microwave was the last to be tested and in many cases yielded the highest temperatures. This tendency of the wax to heat to higher temperatures with subsequent heating was not noted on any of the wax packaging.

#### Definitive

**Figure 1:**
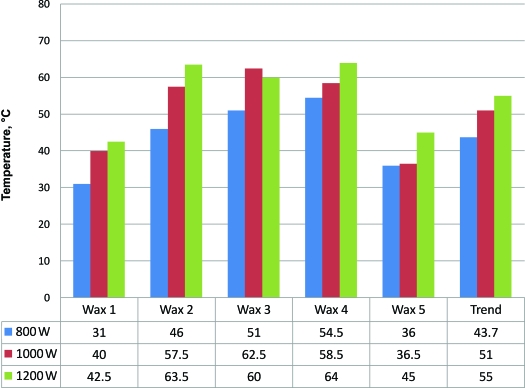
Wax temperatures attained following initial heating of wax (after stirring).

**Figure 2:**
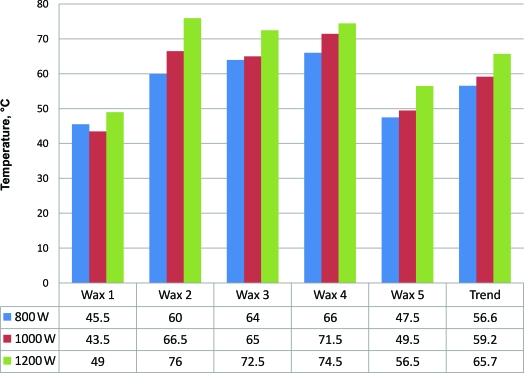
Wax temperatures attained following reheating of wax (after stirring).

**Figure 3:**
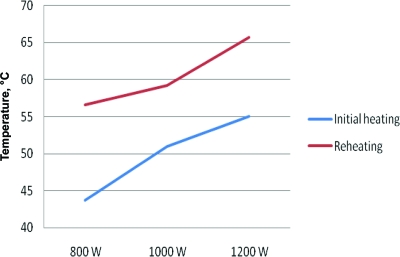
Comparison of wax temperature (after stirring) in microwaves of varying power output when heated and reheated.

The maximum wax temperature recorded at any point was 108.5°C. This occurred before stirring. The maximum temperature recorded from the side of a wax container was 56°C. The maximum wax temperature following stirring was 65.7°C. Of the 60 wax surface temperatures recorded before stirring (central and peripheral for 5 wax products heated and reheated in 3 microwaves), 26 measurements exceeded 70°C. Of those, 22 exceeded 80°C, 17 exceeded 90°C, and 9 exceeded 100°C. Of the 30 stirred wax temperatures recorded, 28 (93.3%) showed an increase in wax temperature with an increase in microwave power output. Wax heated in the 1200-W microwave was on average 9.5% hotter than when heated in the 1000-W microwave. Wax heated in the 1200-W microwave on average was 9.5% and 20.3% hotter than wax heated in the 1000-W and 800-W microwaves, respectively. These results are shown in Figures 1 and 2. Following reheating, the stirred wax was on average 10.7°C hotter than when initially heated (Fig 3).

## DISCUSSION

When undertaking the wax component of this study several modifications were made from the pilot study to ensure comparability of results. During the pilot study, one container of wax from each brand was purchased. Although allowed to cool for more than 16 hours before testing in a subsequent microwave, the same container was heated on each occasion. Our pilot study results showed an increasing wax temperature with the number of times the wax was heated, rather than with increasing microwave wattage. This may be due to decreasing wax content with each heating as wax was removed following stirring, decreasing water content with each heating, or heat-induced alterations in wax properties. During definitive wax testing, 3 of each product were purchased allowing a new product to be tested in each microwave.

In order to eliminate a subjective variable, reheating in the definitive wax study was commenced when the wax cooled to 35°C. Reheating the wax when the user deemed it too viscous for use lead to a variable residual temperature before reheating and might in part account for the lack of correlation between wax temperature and microwave power in the re-reheated pilot study group. Reheating the wax when cooled to 35°C ensured a constant starting temperature, but one that was always greater than the temperature before initial heating. This probably contributed to the reheated wax temperature being consistently higher than that following initial heating. Microwaves of the same brand were chosen during definitive wax testing to increase testing consistency.

The results of this study show that when heating hair removal wax in the microwave according to manufacturer's instructions, wax temperatures regularly reach unsafe working temperatures. As microwave output power increased, wax temperature also increased. Of the 9 wax temperature measurements that reached over 100°C, 8 of them were heated in the 1200-W microwave. Although the stirred wax was not of unreasonable temperatures, this study demonstrated that the wax heats unevenly and some surface areas reach temperatures adequate to cause severe burn injury.

The results must be interpreted within the limitations of the methodology. During this study, each wax was cooled to 35°C before reheating to eliminate a subjective variable; however, many products became too viscous for use before reaching 35°C. In a domestic setting, several of the waxes would have been reheated earlier and higher temperatures would have been attained. The microwaves used in this study were of the same brand but of different models and ages, which may have affected outcome.

From this study, several common microwave-heated hair removal wax product downfalls can be observed. Most contained poor heating instructions. Only one product specified heating instructions for microwaves of different wattages, and several did not specify for what power microwave the instructions were intended. No product instructed the user how to amend heating time and power setting when using a microwave of a different power output. Two products specified ideal wax working temperature, but no way of measuring this. One provided an indicator showing when the wax was too hot, but most contained subjective descriptions such as “thick honey” or “creamy” to indicate when the wax was fit for use. All products indicated that heating times should be reduced with a reducing quantity of wax, but only 2 provided specific heating times, and one of those instructions for microwaves of varying wattage. Four of the 5 products did not contain reheating instructions. This study demonstrated that when reheating is performed according to initial instructions, the wax can become hot enough to cause burn injury.

Four of the 5 products tested warned of the risk of burns from hot wax in the enclosed instruction leaflet, 3 carried burn warnings on the jar, and 1 warned of the risk of burns on the box. One product did not warn of the risk of burn injury. Three of the 5 products contained first aid instructions for burn injury, but only 2 of these described management consistent with current recommendations, the other stating that ice cubes or a cold compress should be used over the burn area.

The data collected from the RAH burns database suggested that many of the burns occurred when removing hot wax from the microwave. This may be partially accounted for by our observation that several plastic wax containers lost rigidity when housing hot wax, and that many of the containers following heating became too hot to hold. Two of the 5 products recommended that wax be heated on a microwave safe plate to reduce the risk of burns from spillage or a hot container.

The results from this study show that the wax heats heterogeneously, as different areas of the wax surface heated to different temperatures, once mixed the temperature dropped significantly. It was observed that areas of wax at a higher temperature were less viscous with a greater tendency to spill; however, only one wax product warned of this.

All waxes required the removal of the lid before heating. Only 2 gave advice regarding what to do in the event that the user forgot to remove the lid. Given that the mean TBSA burnt was 1%, it is possible that the number of patients retrieved from the RAH burns database is not an accurate representation of the incidence of burn injury from microwave-heated hair removal wax. Many who sustain small burn injury may not present to a doctor, be discharged directly from the emergency department, or be treated by a primary care practitioner rather than a hospital and would not have been included in the collected data.

We have recently learned that the South Australian Government Department of Health (following a short television segment about our experiences) has indicated that they plan to investigate this issue more deeply. This may result in legislative change.

## CONCLUSION

Microwave-heated hair removal wax has the potential to reach unsafe temperatures and cause burn injury even when manufacturer's heating instructions are followed. All hair-removal wax products in this study included instructions for heating but most were inadequate for safe use. To safely use hot hair-removal wax in a domestic setting, heating instructions need to be improved. Given that the majority of burns resulted from wax spillage during removal from the microwave, the packaging of all microwave-heated hair removal wax products should warn that, following heating, the surface wax will become hotter and less viscous than the wax below, and the container may become less rigid and too hot to hold. This should be accompanied by a recommendation that wax is heated on a microwave-safe plate to reduce spillage, and the wax be stirred before use. Heating instructions should include appropriate microwave wattage for heating, an indicator when the wax is too hot for use—for example, a thermometer or color indicator—and detailed instructions for heating and reheating when the container is of varying fullness. Before the most common injury involved the hands (76%), additional safety instructions suggesting that the hot product is removed from the microwave using “oven gloves” might prevent most injuries. Also included should be a warning of the risk of burn injury and simple but appropriate first aid instructions. Most burn injury caused by hair removal wax is preventable, but requires user awareness as well as appropriate warnings and first aid instruction to be provided.

## Acknowledgment

The authors thank Sean Jolly for his assistance graphing the data.

## Figures and Tables

**Table 1:** Data from patients extracted from the Royal Adelaide Hospital burns database with burn injury resulting from hot hair removal wax

	Age	Site	Depth	TBSA (%)	Length of stay	Management
1	26	• Right arm	Superficial partial/Mid-dermal	3	8	DUA + Biobrane
		• Left arm				
		• Right thigh				
2	19	• Bilateral thighs	Superficial partial	1	2	DUA + Biobrane
3	18	• Left hand	Superficial partial	2	3	DUA + Biobrane
		• Bilateral thighs				
4	19	• Right hand	Mid-dermal	1	3	DUA + Acticoat
5	32	• Left hand	Superficial	1	1	Nil
6	44	• Right hand	Superficial	1	2	Nil
		• R foot				
7	18	• Left thigh	Mid-dermal D	1	2	Nil
8	20	• Left hand	Superficial	1	1	Nil
9	27	• Right hand	Superficial	1	0	Nil
10	41	• Left calf	Full thickness	1	4	DUA + grafting
11	30	• Left hand	Superficial	2	1	Nil
12	21	• Chest	Partial	1.3	5	DUA + Biobrane
		• Both hands				
13	51	• Both hands	Superficial partial/Mid-dermal	1.5	4	DUA + Biobrane
		• Left foot				
14	16	• Right hand	Mid-dermal	1	1	Nil
15	19	• Face	Mid-dermal	1	1	Nil
		• Left hand				
16	15	• Right hand	Deep dermal	1	4	DUA + grafting
17	17	• Right hand	Mid-dermal	1	4	DUA + Biobrane
18	20	• Right hand	Unknown	Unknown	1	DUA + Biobrane
19	54	• Face	Superficial	1	3	Nil
20	28	• Right hand	Superficial	1	0	Nil
21	21	• Both hands	Superficial Partial	1	2	Nil
